# Ti/CuO Nanothermite Doped with Secondary Energetic Materials: A Study of Combustion Parameters

**DOI:** 10.3390/molecules29153664

**Published:** 2024-08-02

**Authors:** Mateusz Polis, Agnieszka Stolarczyk, Konrad Szydło, Barbara Lisiecka, Marcin Procek, Sebastian Sławski, Wojciech Domagała, Jakub Iksal, Tomasz Jarosz

**Affiliations:** 1 Łukasiewicz Research Network—Institute of Industrial Organic Chemistry, Explosive Techniques Research Group, 42-693 Krupski Młyn, Poland; 2 Department of Physical Chemistry and Technology of Polymers, Silesian University of Technology, 44-100 Gliwice, Poland; 3 Department of Optoelectronics, Silesian University of Technology, 2 Krzywoustego Str., 44-100 Gliwice, Poland; 4 Department of Theoretical and Applied Mechanics, Silesian University of Technology, 18A Konarskiego Str., 44-100 Gliwice, Poland; 5 Student at the Faculty of Chemistry, Silesian University of Technology, 44-100 Gliwice, Poland

**Keywords:** NSTEX, nanothermite, energetic material, combustion, sensitivity

## Abstract

Nanothermites have found broad applications; however, due to being systems largely reacting in condensed phases, their performance is somewhat limited by heat and mass transfer. In order to alleviate this issue, nanothermites doped with gas-generating energetic materials have been developed. In this work, we present an investigation of a model Ti/CuO nanothermite doped by four classical energetic materials and investigate their properties and combustion performance. Mechanical and laser irradiation sensitivity, as well as ignition/explosion temperatures have been determined for the studied systems to establish their safety features. In terms of combustion performance, thrust force parameters and linear combustion velocity have been determined and the structure of the evolving flame front was recorded during open-air combustion experiments. The obtained results indicate that the developed doped nanothermite formulations are extremely promising materials for future applications.

## 1. Introduction

Nanothermites (NT) or metastable interstitial composites (MIC) [1] are pyrotechnic compositions consisting of fuel or reducing agent and oxidising agent, with particle dimensions on the order of nanometers [2]. The reducing agent (fuel) is typically a metal, such as Mg, Al or Ti and the oxidising agent is typically a metal oxide, e.g., CuO or Fe_2_O_3_ [3]. In comparison with thermite compositions, whose components are micrometric in size, nanothermites are typically more homogeneous, exhibiting lower combustion induction times, high reaction heats and higher combustion velocities [4], typically up to 1000 m/s [5,6,7]. Better contact between the fuel and the oxidising agent also results in extremely high reactivity, resulting in high sensitivity to initiating stimuli. Nanothermite compositions are used in the production of initiators to replace lead-based energetic materials, such as lead styphnate [8], in welding systems [9] and in microthrusters [10].

Among nanothermites, Al-based systems are most commonly investigated, with Al/CuO being perceived as a nigh-model system, even though many facets of its properties and combustion mechanism are still being uncovered [11]. Despite this popularity, the use of various fuels, such as magnesium-aluminium alloys [12] or titanium [13] has recently been reported, including nanothermites for special applications, such as Ti/WO_3_.

Nanostructured thermites and explosives (NSTEX) are systems produced by supplementing a nanothermite with a secondary energetic material (EM) [14]. Due to the addition of the EM, NSTEX properties differ from those of nanothermites. Of particular interest is the increase in achievable combustion velocities and a more violent nature of their combustion [15]. Among the EMs used to produce NSTEXs, 1,3,5-Trinitro-1,3,5-triazinane (RDX), cellulose nitrate (NC) and 2,4,6-trinitrotoluene (TNT) are commonly used [16]. Recent works have suggested the development of NSTEXs using propano-1,2,3-triol trinitrate (“nitroglycerin”) trapped in melamine matrices [17] and even energetic coordination compounds [18]. NSTEXs have found initial application in micro-electronic mechanical systems (MEMS) [19], multifunctional MIC chips [1] and in microthrusters [20].

Both nanothermites and NSTEX compositions can be used in microthrusters. Such systems are indispensable in spacecraft applications in order to realize precision altitude control [21]. Due to the need to ensure low response times and short thrust duration in microthrusters, it is advantageous to use nanothermites or NSTEX as propellants. Compared with other micropropulsion systems, these microthruster systems are smaller, lighter, simpler and more reliable [21]. Additionally, it is possible to change the specific impulse, depending on the needs, by changing the content of additional EM [20]. NSTEX compositions are the best option to use in microthrusters, because of the combination of advantages of their ingredients. As a result, they have low response times and short thrust duration due to the nanothermites and high burning velocities due to the additional EMs.

The main idea of this work is to investigate the impact of several EMs on the parameters of the Ti/CuO-based system in the context of potential propulsion applications. The selected energetic materials are NTO (5-Nitro-1,2-dihydro-3H-1,2,4-triazin-3-one), PETN (2,2-Bis[(nitrooxy)methyl]propane-1,3-diyl dinitrate), RDX (1,3,5-Trinitro-1,3,5-triazinane) and HMX (1,3,5,7-Tetranitro-1,3,5,7-tetrazocane). The resulting compositions are denoted as NT-N (nanothermite doped with NTO), NT-P (nanothermite doped with PETN), NT-R (nanothermite doped with RDX) and NT-H (nanothermite doped with HMX). Due to this motivation, it is necessary to examine the parameters such as the thrust force, sensitivity on initiation stimuli and combustion velocity of these NSTEX compositions.

## 2. Results and Discussion

### 2.1. SEM-EDS

**Figure A1:**
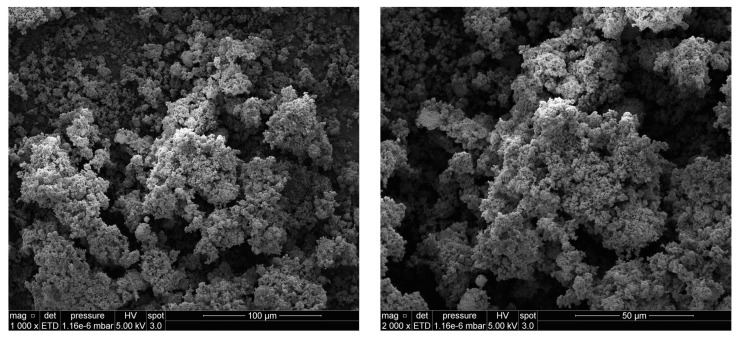

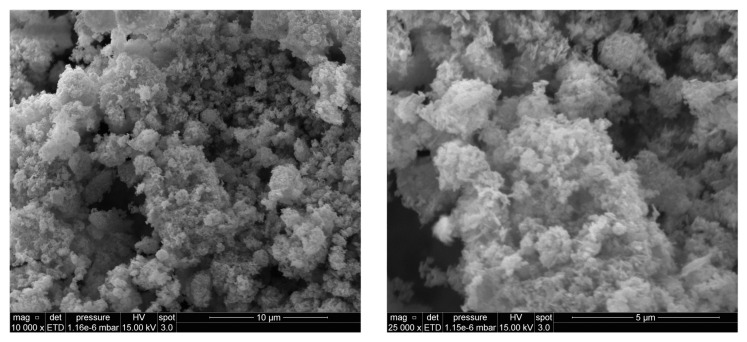
SEM images of NT-N composition.

**Figure A2:**
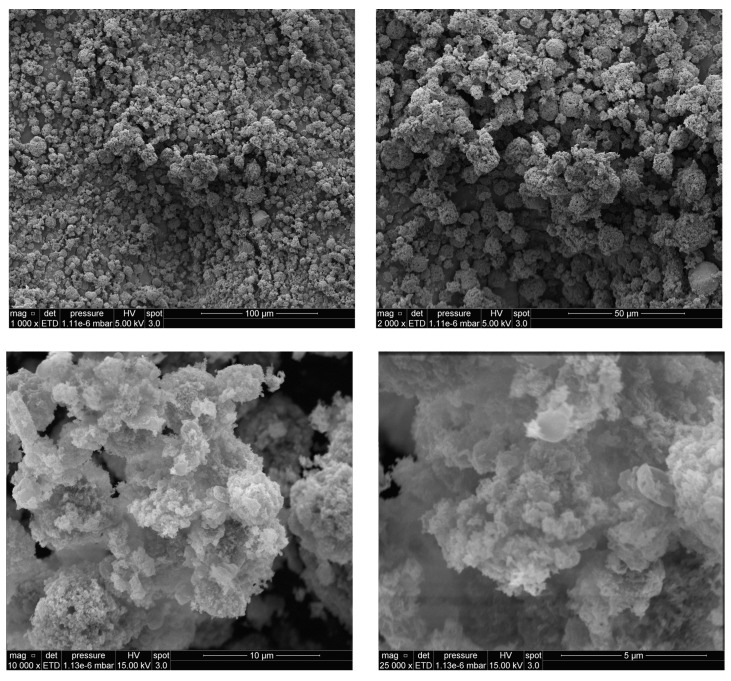
SEM images of NT-H composition.

**Figure A3:**
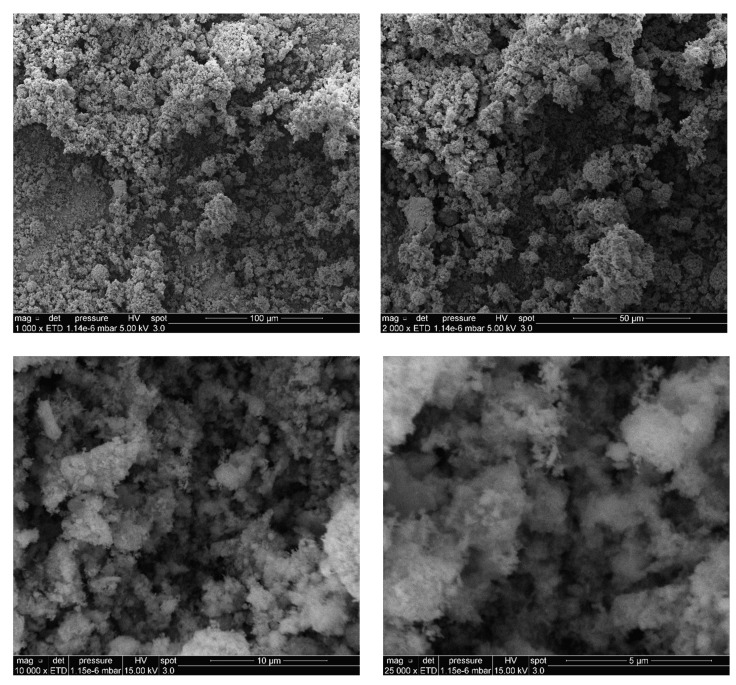
SEM images of NT-P composition.

**Figure A4:**
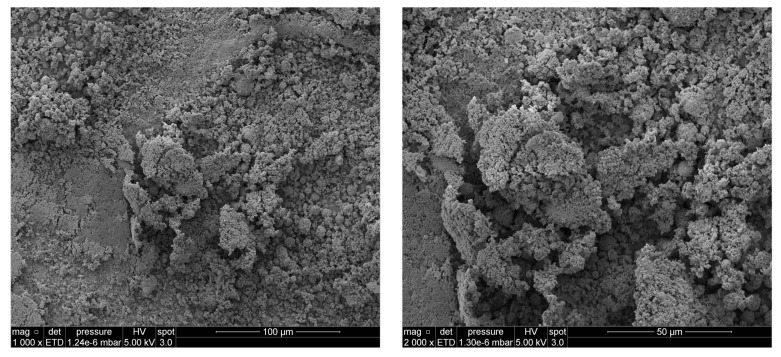

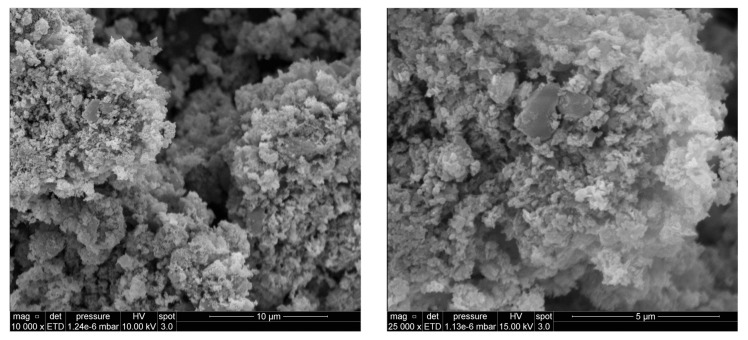
SEM images of NT-R composition.

The SEM images of NT-N sample (Figure A1) confirmed the presence of large, micrometric agglomerates with uneven surface and composed of smaller, spherical and multi-walled particles. Except for large agglomerates, the presence of smaller, fluffy and porous particles can be pointed out. Importantly, the spread of particle size is fairly wide. In the case of the NT-H sample (Figure A2) the sample contains mostly regular, large, quasi-spherical particles covered with small amounts of submicrometric, irregular crystals with multi-walled or even spindle geometry. Nevertheless, the homogeneity of the whole composition in terms of particle size and shape is higher than for the NT-N composition. The analysis of NT-P confirmed slightly different morphology (Figure A3). Still, we may point to the presence of larger agglomerates and particles, but a significant part of the sample is made up of submicrometric grains with an irregular structure. The NT-R composition (Figure A4) showed the best morphology among the tested samples. In SEM images, we can observe the presence of mainly small, submicron particles of varying shapes, assembling into individual larger agglomerates.

In terms of assessment of SEM analysis results, we can connect the strong differences in morphology of tested NSTEXs with fairly different solubility of used EMs. In temperature of 25 °C the solubility in acetone of RDX, PETN and HMX are equal to 61.41 g/L [22], 249.5 g/L [23] and 19.30 g/L [22], respectively, whereas the solubility of NTO in isopropyl alcohol is equal to 15.75 g/L [24]. The large differences in solubility of EMs affected the electrospraying process, compounds with lower solubility crystallised faster during the process. At the same time, the acetone is characterised by much higher vapour pressure than isopropyl alcohol, which results in faster evaporation of the solvent during the process. Due to the factors discussed above, the NT-R and NT-P compositions exhibit the expected morphology.

In the case of the SEM-EDS results, for the NT-N sample, the Cu atoms are distributed equally in the whole sample except for areas with increased Ti content, where these areas are most likely micrometric fuel grains. At the same time, the rest of the Ti atoms are distributed fairly uniformly. Even though the O atoms are deployed similarly uniformly, for the C and N atoms we can point out areas with higher concentrations, most likely related to the presence of larger NTO crystals. In the case of the NT-P sample, the distribution of Cu and Ti atoms is similar, with micrometric fuel grains being observed. However, the sample has areas showing lower Ti content. O, C and N atoms are located fairly evenly, with slightly richer areas along with Ti. Unlike in NT-N, no large single EM crystals can be found. Instead, evenly distributed small, submicrometric PETN grains are observed. The NT-H sample shows poor homogenisation. The distribution of Ti atoms is uneven and except for large micrometric fuel grains, nanometric Ti agglomerates are present as well. Similar to NT-P, the areas rich in C and N atoms (HMX) are distributed in areas with increased Ti content. In the case of the NT-R sample, analogous conclusions can be drawn.

### 2.2. Laser Sensitivity

**Figure 1:**
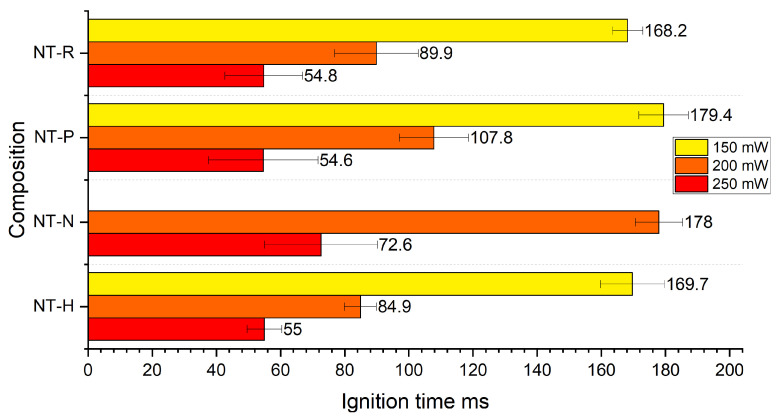
Ignition time of tested compositions for varied radiation powers.

The laser sensitivity measurement results are shown in Figure 1.

All tested compositions showed tunable relation between radiation power and ignition time. With decreasing radiation power, a fast increase in ignition time is observed. This is significant enough that for radiation powers of 150 mW (for NT-N) and 100 mW (other samples), no ignition is observed. For the highest utilised irradiation power (250 mW), the ignition time was very similar for all tested NSTEX samples (variation of up to 10 ms). Irradiation with 200 mW resulted in the ignition times becoming more visibly dependent on the type of sample. The longest ignition times were achieved for NT-N for all utilised radiation powers. This likely stems from the properties of the EM that was used as the additive, i.e. its high critical diameter, low reactivity in comparison with other EMs and high decomposition temperature.

Two main factors need to be taken into account when considering the experimental results. First, the radiant energy absorption of the NSTEXs may vary to an extent, due to the different absorption coefficients of their components (i.e. the introduced EMs). In order to estimate absorption capacity of the tested compositions, initiated with a laser (λ = 520 nm), a measurement of laser reflection for the raw materials used in this work and for the NSTEX samples was conducted. Tests analogous to those described in literature [25] have been performed. In the cited work, the use of photoelectric sensors to detect reflected light and measure reflectance was reported. It was found that the average laser reflectance is an acceptable measure of laser absorption of the compositions.

**Figure 2:**
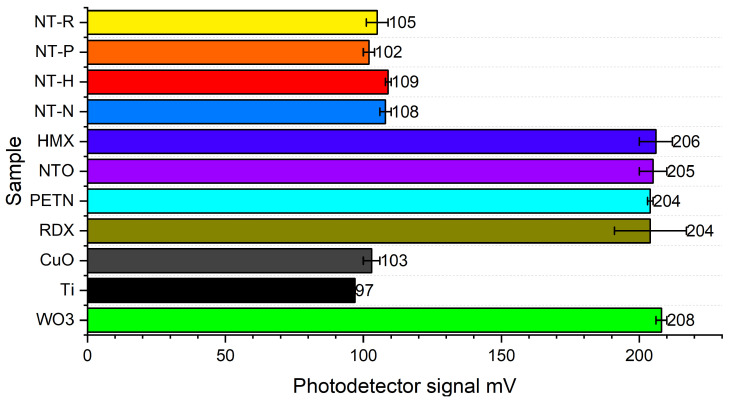
The reflected light intensity of samples. The reference is tungesten(VI) oxide sample.

Each component of the tested compositions was exposed to laser radiation (520 nm, 50 mW) and the signal of the reflection from the sample surface radiation was measured by the photodetector. The results of tests are presented in Figure 2.

The reflected light intensity is relatively high for the tested EMs, with the individual compounds differing only slightly. Moreover, the signal of the photodetector shows (Figure 2) that the reflected light intensity of EMs was slightly lower than that of the tungsten(VI) oxide, which was part of the control group. This indicated that the EM crystals had poor laser absorption. This is due to the fact that organic compounds have significant absorption coefficients in the mid-infrared region (2.5–25 μm), which contains the main bands of their vibrational spectra [26]. In contrast, Ti and CuO were found to reflect much less light, which showed that these materials had a stronger laser absorption capacity than the EM crystals. Similar photodetector signals were obtained for the tested NSTEX samples. Moreover, the compositions have similar laser absorption capacity with respect to CuO, which is related to the morphology of the samples and the higher mass proportion of Ti and CuO than EMs in the sample.

Consequently, the EMs can be viewed as decreasing the ability of the NSTEXs to absorb laser light. Secondly, the used EMs are characterised with similar thermal conductivity coefficients, significantly lower than Ti and CuO. However, as proven via AET tests, the compositions are characterised by a low ignition temperature.

### 2.3. Friction and Impact Sensitivity

The friction and impact sensitivity were tested both for NSTEXs compositions and pure EMs playing the role of additive to NT composition (Table 1). One can notice that except for NT-P composition, the impact sensitivity dipped down rapidly. The fairly effect on PETN containing NSTEX is most likely related to the small critical diameter of PETN (1.5 mm [27]), high impact sensitivity of pure PETN and morphology of the obtained composition. Notwithstanding, the decreasing effect on sensitivity is related to the high content of NT in the tested compositions, which is characterised by a low impact sensitivity.

The friction sensitivity results point to more complex dependencies. In the case of NT-H and NT-N a fairly significant increase in measured value is seen. This effect results from the presence of solid components characterised by high hardness (in comparison to EMs) and the morphology of the prepared compositions, as discussed in the SEM section. Also, the catalytic effect of nano-CuO and nano-TiO_2_ (present as an oxide layer on the surface of the fuel particles) on the decomposition process of EMs should also be taken into account [28,29]. Conversely, for NT-P and NT-R the effect is opposite, which stems both from ultrafine EMs particle size, as the decrease in the particle size of EMs leads to lower sensitivity towards mechanical stimuli [30] and good component distribution as well.

### 2.4. Ignition/Explosion Temperature

**Figure 3:**
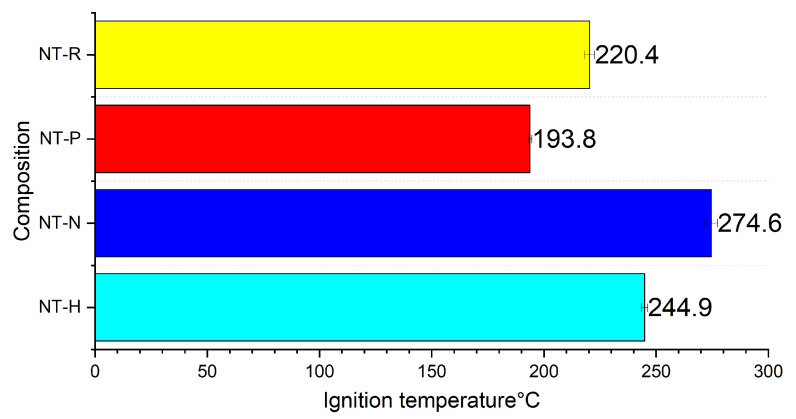
Ignition temperature of tested compositions.

The ignition temperature measurement results are presented below (Figure 3).

The effect of NT on the EM decomposition temperature is clearly visible for three of the tested compositions. The decomposition temperatures of RDX, PETN, NTO and HMX are 245.6 [31], 208.3 [32], 276.4 [33] and 285.4 [34] respectively. The highest difference between ignition values is recorded for the NT-HMX sample (14.2% lower ignition temperature). The observed effect may result from the catalytic effect of nano-CuO or nano-TiO_2_ on the decomposition of the used EMs [28,29]. The lack of a catalytic effect of CuO nanoparticles on NTO decomposition is in line with other results reported in literature [35], but the influence of nano TiO_2_ on ignition temperature was confirmed in the same work [35]. Notwithstanding, the TiO_2_ may be present only as a passive layer on the fuel core, along with other titanium oxides (as TiO, Ti_2_O_3_). Thus, the concentration of TiO_2_ can be insufficient to impact ignition temperature.

### 2.5. Thrust Force

**Figure 4:**
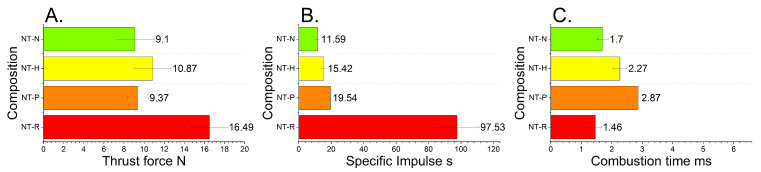
The maximum thrust force (**A**), specific impulse (**B**) and combustion time (**C**) of tested compositions.

The results of thrust force measurements are presented in Figure 4.

Among the tested samples, the NT-R is characterised by the best parameters, the highest thrust force, impulse and the lowest combustion time. Nevertheless, we can point to significant differences between the behaviour of the tested compositions. Sorting the compositions in decreasing thrust force order, the sequence NT-R, NT-H, NT-P and NT-N is obtained. Simultaneously, sorting by maximum impulse results in the sequence of NT-R, NT-P, NT-H and NT-N. This dissimilarity results from much longer combustion times of NT-H and NT-P. In such a small scale (15 mg samples), the effects related to composition morphology could play a predominant role. The discrepancy between combustion times for this test and open-air test stems from the small mass of the sample (15 mg) and performing tests for the bulk density of samples.

### 2.6. Combustion Velocity

**Figure 5:**
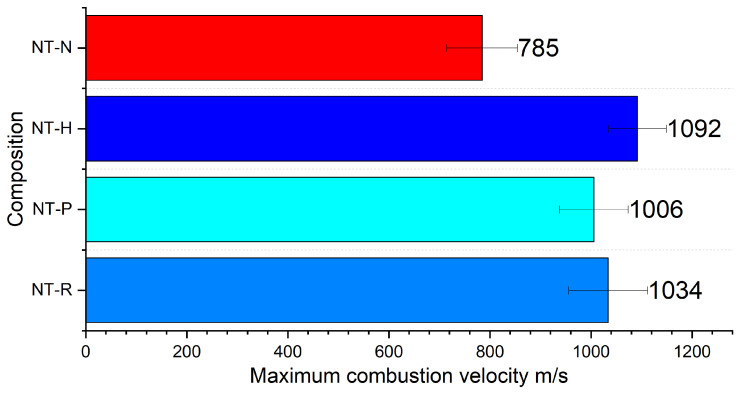
Maximum combustion velocity of tested compositions.

**Figure A13:**
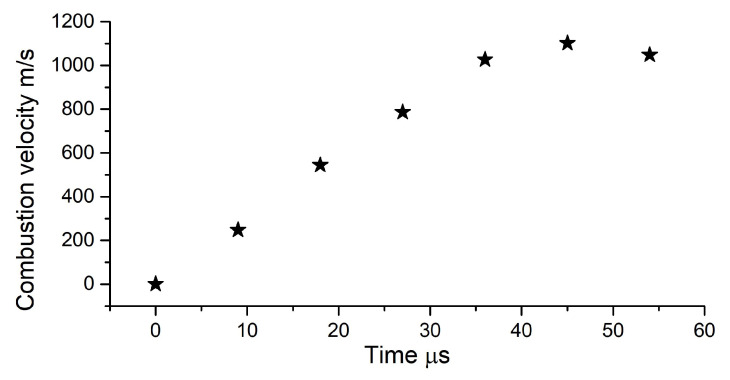
Combustion velocity vs. time plot recorded for NT-R sample combustion.

Taking into account all tested compositions, it can be seen that they showed similar behaviour (Figure 5). After ignition, the combustion process undergoes rapid acceleration up to fairly stable maximum combustion velocity (Figure A13). The similar maximum combustion velocities for PETN, RDX and HMX-based NSTEX indicates the slight impact of the EM in the composition on this parameter. Nevertheless, the significantly lower value for NT-N composition most likely results from NTO exhibiting a higher critical diameter than the other used EMs [36]. The effect of EM choice will be more pronounced if a higher EM content in relation to nanothermite is employed.

**Figure 6:**
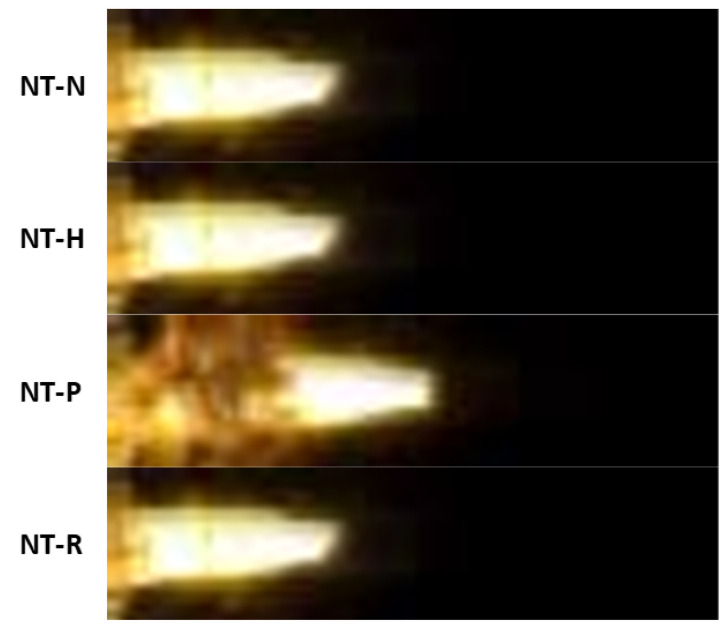
Snapshot of combustion of tested samples 45 μs after ignition.

The achieved values point to the occurrence of a energetic process in the form of rapid deflagration rather than detonation. Moreover, the registered images (Figure 6) depict the flame front with a complex, shattered structure rather than an approximately flat detonation front. Nevertheless, one should notice the scattering of the PMMA cover during propagation, which could affect the combustion development and maximum sampling rate of the used high-speed camera.

The results may lead to an interesting conclusion. For the tested NSTEXs with quite a small content of EMs (15 wt. %), the choice of EM does not affect the propagation mechanism. The EM acts as a gas generator, providing sufficient amount of gases for the combustion mechanism limited by mass and heat transfer. With the high NT content in the tested composition, the heat transfer in the solid or liquid phase could still play an important role. At the same time, the presence of solid compounds with high melting and boiling points (Ti-CuO ) will consume huge amounts of heat, therefore, limiting the combustion parameters. The highest combustion velocity for NT-H sample derivatives from the highest high-energy parameters among used EMs-eg, detonation velocity, heat of detonation or gas production.

### 2.7. Open-Air Combustion Tests

**Figure 7:**
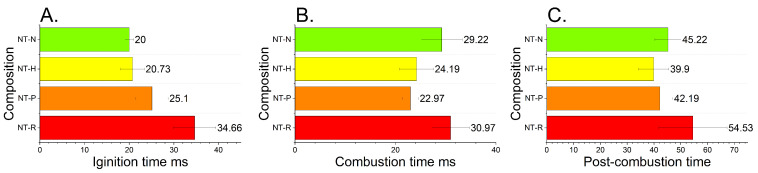
The results of combustion tests with a distinguished ignition time (**A**), main combustion time (**B**) and post-combustion time (**C**).

The open-air combustion tests have revealed three main phases of combustion of the samples. The ignition phase, the main combustion phase and the post-combustion phase can be observed. The analysed phases are shown in Figure 7. In the first stage, a concentrated flame is observed, the shape and intensity of which change depending on the EM used in the composition. In the next phase, the expansion of the flame and the appearance of sparks can be observed, which is caused by the release of gases. Depending on the utilised EM, this process is more or less rapid. In the post-combustion phase, larger particles of the reacting composition dispersed from the plate undergo combustion.

**Figure 8:**
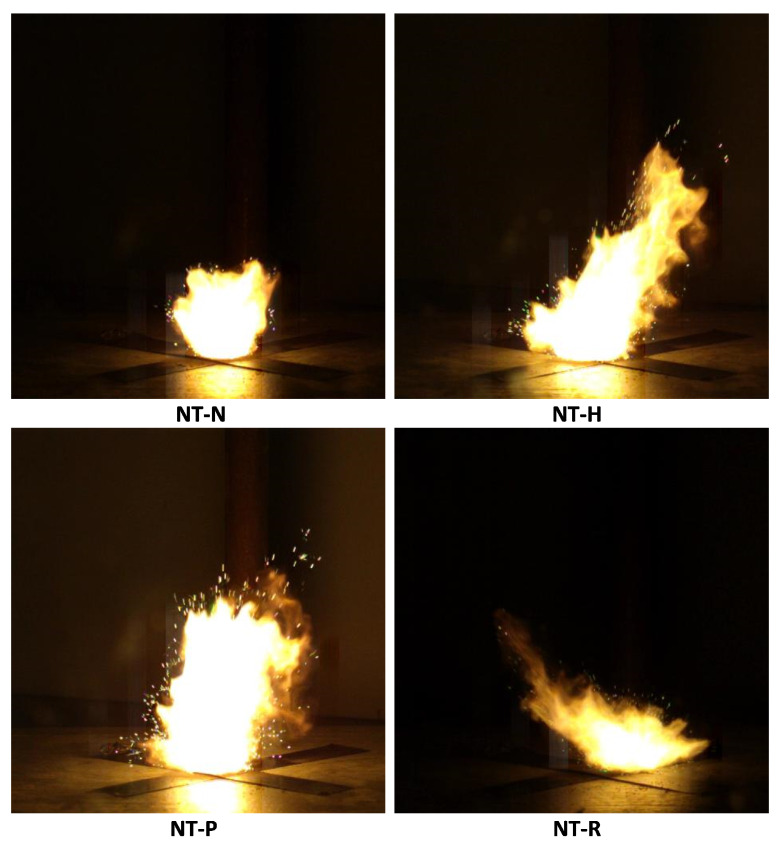
Snapshots of combustion of tested samples 9.6 ms after ignition.

The highest ignition time was recorded for the NT-N composition. For the rest of the samples tested, the ignition times are fairly similar to each other. The results of open-air tests are in line with laser sensitivity tests, and the fairly constant ignition time for all compositions except for NT-N with much lower radiation sensitivity. The small differences may result from the methodology of the test, in which the tested material is poured without any cover onto the ceramic plate and ignited. Therefore, the different geometry of the sample between tests may affect results. The duration of the main stage of combustion is the shortest for the NT-H composition and the longest for the NT-N composition. In the case of the NT-R sample, despite achieving the shortest ignition time, the duration of the main phase is longer compared to NT-P and NT-H. The combustion process becomes more rapid for samples containing HMX and PETN (Figure 8). The development of the process is faster and more sparks can be seen. This is associated with the formation of a large amount of gaseous products that cause the acceleration of the combustion process. In the case of compositions containing RDX and NTO, a lower number of sparks can be observed in the main combustion stage. This indicates that most of the combustion reactions occurred inside the flame zone, and the scale of solid phase reactions increased.

**Figure 9:**
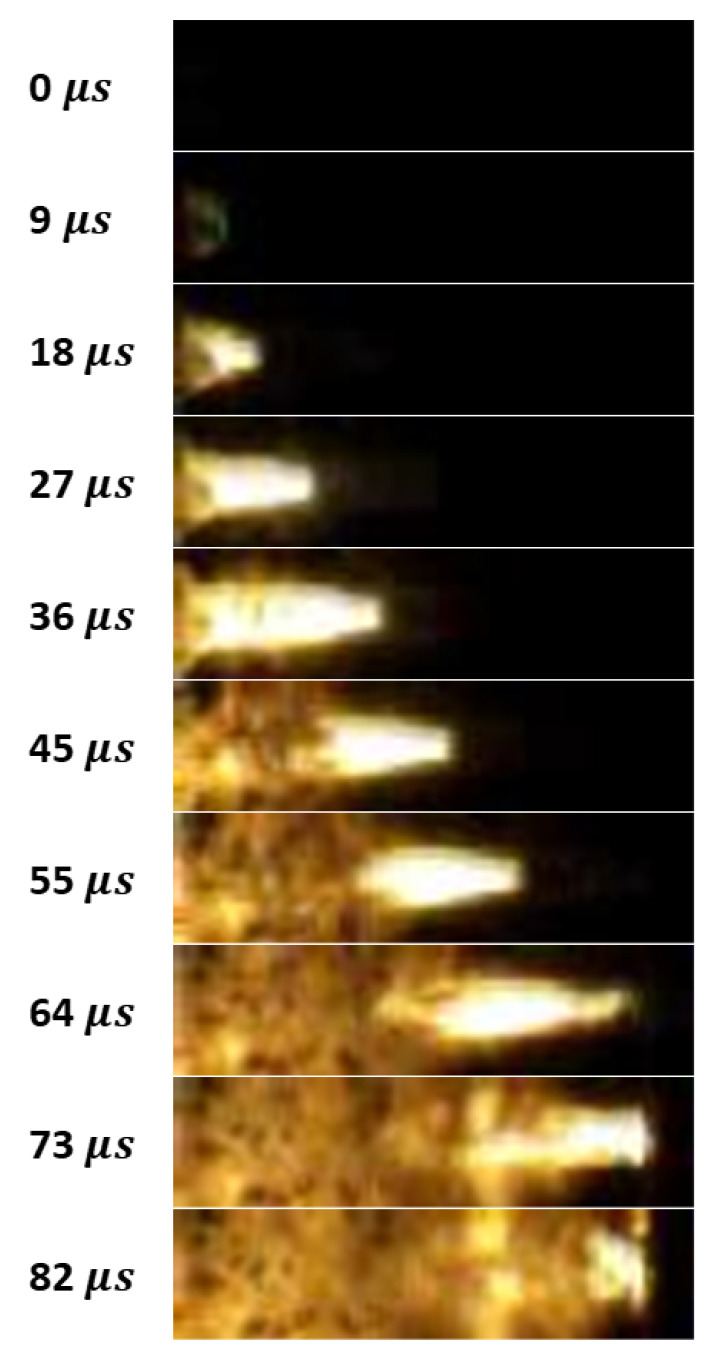
Snapshot of combustion of NT-P sample.

The post-combustion stage is the longest for NT-N compositions, yet a rather large measurement error must be taken into account. Whereas there is an apparent shortening of the post-combustion time for NT-P compared to the rest of the samples. Analysing all phases of combustion, it was found that the addition of NTO to nanothermites slows the ignition and combustion development of the tested compositions (Figure 9).

## 3. Materials and Methods

### 3.1. Materials

All used materials are listed below. All materials were used as received, without purification.

- Titanium nanopowder, 99.9% purity, average particle size equal to 50 nm (Iolitec GmBH, Heilbronn, Germany);
- Copper (II) oxide, 99.99% purity, average particle size equal to 40 nm (Iolitec GmBH, Germany);
- 4% Collodion solution (Sigma-Aldrich, Darmstadt, Germany);
- Propan-2-ol, 99.9% purity (Electro Chem, Puszczykowo, Poland);
- Acetone, 99.9% purity (Chempur S.A., Piekary Śląskie, Poland);
- 5-Nitro-1,2-dihydro-3H-1,2,4-triazin-3-one (NTO), 99.5% purity, average particle size equal to 200 μm (Nitrochem S.A., Bydgoszcz, Poland);
- 2,2-Bis[(nitrooxy)methyl]propane-1,3-diyl dinitrate (PETN), Class 4 according to MIL-P-387 C (Nitroerg S.A., Poland);
- 1,3,5-Trinitro-1,3,5-triazinane (RDX), Class 5 according to MIL-DTL-398D standard (Nitrochem S.A., Poland);
- 1,3,5,7-Tetranitro-1,3,5,7-tetrazocane (HMX), Class 1 according to MIL-DTL-45444C standard (Nitrochem S.A., Poland);
- Tungsten(VI) oxide, 99.99% purity, average particle size of 55 nm (Nanografi Nano Teknoloji AS, Ankara, Türkiye).

In order to examine the effect of EM additive on NSTEX system, compositions with the addition of NTO (NT-N), RDX (NT-R), HMX (NT-H) OR PETN (NT-P) were prepared (Table 2). The content of prepared compositions is presented below.

The compositions were prepared on the base of Ti/CuO nanothermite with equivalence ratio (ϕ) equal to 1.4. In first step, the collodium solution was dried out at a temperature of 90 °C for 6 h to obtain pure NC. In the next step, all components were weighed after the drying process (60 °C, 12 h), transferred into bottles and filled with solvent (propan-2-ol in case of NT-N, acetone was used as solvent for the rest of EMs) in such a manner as to achieve solid loading of 125 mg/mL. The prepared suspensions were mixed using a magnetic stirrer for 30 min, sonicated for 30 min (UH50 sonicator, 30/70 break/sonication cycle) and stirred for the next 48 h in order to achieve stable suspensions. In the next step, the suspensions were transferred in small portions (1 cm^3^) into syringes and electrosprayed (distance between nozzle and collector, 10 cm, voltage difference, 19 kV, nozzle internal diameter, 0.5 mm, flow rate, 3 cm^3^/h). After the electrospraying process, the compositions were gently removed from collectors with a spatula, dried (60 °C, 12 h) and closed in sealed containers.

### 3.2. SEM-EDS

The chemical composition and morphology of tested compositions were examined with an FEI Inspect S50 (FEI, Hillsboro, OR, USA) scanning electron microscope (SEM), coupled with X-ray energy dispersive spectrometer with Detector EDS Octane Elect Plus and Analyzer EDAX Z2-i7 (Bruker, Billerica, MS, USA), enabling simultaneous acquisition of micrographs and Energy Dispersive X-ray (EDS) maps of the investigated samples. The authors used the following parameters: working distance of 10 mm, acceleration voltage of the incident electrons was in fhe range of 5–15 kV, with intensity of ca. 95 μA, and the spot size of the electronic beam equal to 3.

### 3.3. Laser Sensitivity

The laser sensitivity tests were performed for radiation wavelength equal to 520 nm and radiation power in the range of 100–250 mW. The measurements were carried out with a laser pulse generator Laser Green 520-1000-SMA (Spectralaser, Opole, Poland). Before each measurement, the laser power was tested with the MIER-4W-USB thermal radiation power detector (SpectraLaser, Poland). The ignition process of samples was recorded using a photodetector working in UV-Vis (350–800 nm) range. Laser generator and photodetector were connected with Tektronix TBS2401B digital oscilloscope, Tektronix, Beaverton, OR, USA with a sampling frequency of 1 MSa/s. For each experiment, 15 mg samples of tested compositions were poured into a copper shell positioned by an alumina ring with a cavity. The radiation impact area was equal to 2.2 mm^2^. Tests were repeated at least 10 times for each composition and radiation power.

### 3.4. Friction and Impact Sensitivity

All tests were performed in accordance with the relevant international standards. The friction sensitivity test was conducted using Peter’s apparatus according to the standard [37]. Prior to measurement, the 40 μL (approximately 25–35 mg) of the sample was placed in the form of a thin layer between the ceramic cylinder and the ceramic plate. By changing the suspended weight and the place on the apparatus arm where it was suspended, the authors regulated the friction force. The sample reaction was observed through sound, smoke appearance, or by the characteristic smell of the decomposition products. The lowest the normal force value at which the sign of reaction was noticeable in at least one of six trials is reported as the friction sensitivity (lower sensitivity threshold).

The impact sensitivity test was carried out with BAM Fallhammer Apparatus. The impact energy of a falling drop weight of known mass was determined by varying the height from which it falls and the drop weight according to the standard [38]. For this measurement, 40 μL (approximately 25–35 mg) of each sample was placed centrally between two metallic cylinders fixed with a steel sleeve. The sample initiation was observed through sound, smoke appearance, or by the characteristic smell of the decomposition products. The lowest the impact energy value at which the sign of reaction was noticeable in at least one of six trials is reported as the impact sensitivity (lower sensitivity threshold).

### 3.5. Ignition/Explosion Temperature

The ignition/explosion temperatures (IETs) of prepared compositions were determined using an Automatic Explosion Temperature 402 Tester (OZM Research, Hrochův Týnec, Czech Republic), for 50 ± 1 mg samples. The measurement was carried out in the range of 100–400 °C, for heating rate equal to 20 K/min. Measurements for each sample were repeated at least three times.

### 3.6. Thrust Force

**Figure 10:**
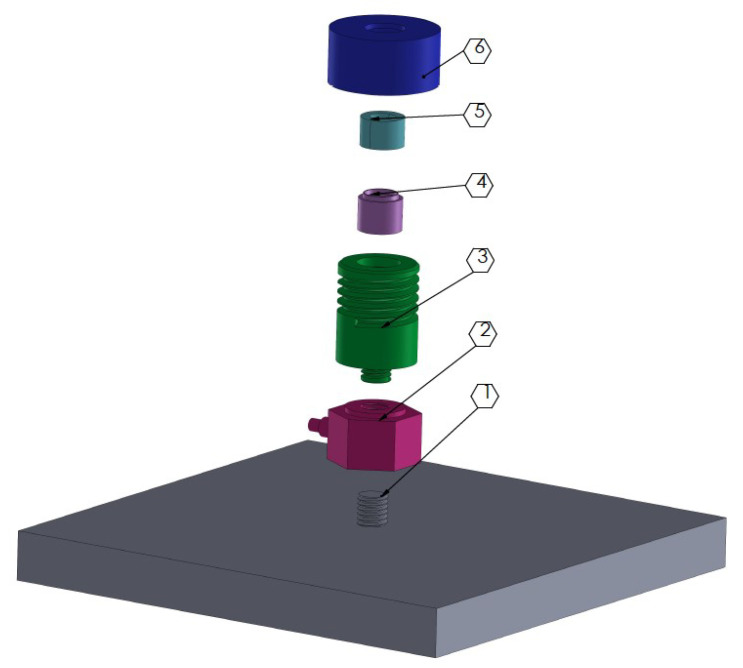
Scheme of thrust measurement set-up, where: 1—steel base, 2—force sensor, 3—threaded connector, 4—shell, 5—nozzle, 6—locking nut.

The scheme of the set-up used to measure the thrust force is presented below (Figure 10).

Fifteen milligrams of the samples of tested compositions were dried for 12 h at 60 °C before the tests. In each test, the sample of the tested composition was transferred into a 3D printed shell (printed from Noctuo ABS filament with a Prusa Mk3 3D printer, Prusa, Prague, Czech Republic) and closed with 3D printed convergence-divergence nozzle (printed from Noctuo ABS filament with Prusa Mk3). In the next step, the prepared charge was mounted inside a tailored cavity using a steel connector, attached to the force sensor (PCB Piezotronics 208C02, PCB Piezotronics, Depew, NY, USA), which was coupled to a steel base plate. The charge was blocked with a locking nut. Above the nozzle, the optical collimator connected with a laser pulse generator Laser Green520-1000-SMA (Spectralaser, Poland) was placed in such manner, as to facilitate ignition of the sample. Before each measurement, the laser power was tested with the MIER-4W-USB thermal radiation power detector (SpectraLaser, Poland). The samples were ignited by a radiation pulse (700 mW, 100 ms). The force sensor (PCB Piezotronics 208C02) was connected to a signal conditioner (PCB Piezotronics 480E09) and to a digital oscilloscope (Tektronix TBS2401B). All collected data were analysed using OriginPro, version 2022 software. Tests were repeated at least 10 times for each composition.

### 3.7. Combustion Velocity

**Figure 11:**
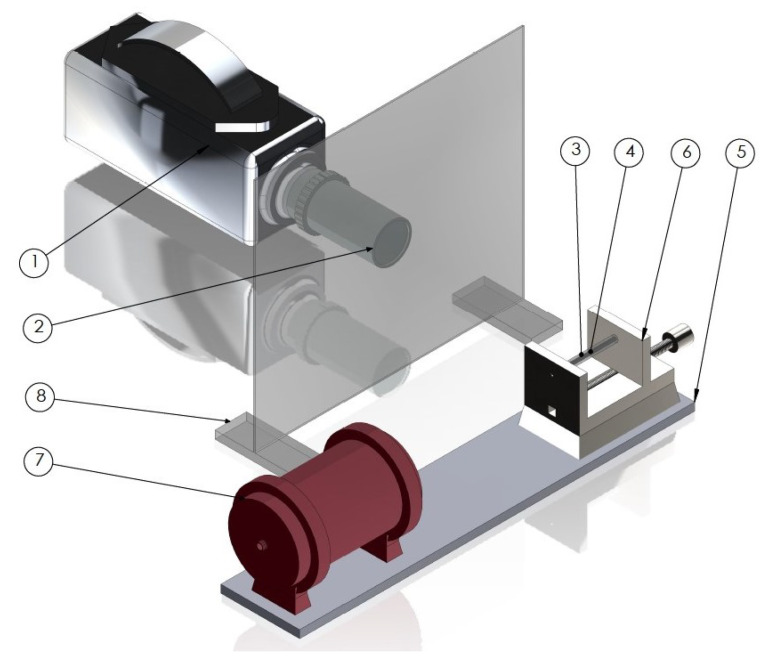
Scheme of open-air test set-up, where 1—high-speed camera; 2—optical lens, 3—PMMA tube, 4—nanothermite sample, 5—basic plate, 6—mounting plates, 7—optical collimator, 8—PMMA shelter.

In order to measure the combustion velocity, the tested compositions were elaborated into PMMA tubes (4 × 2 mm in diameter, 70 mm long) terminated with PMMA window at one end, and closed with 3D printed plug (printed from Noctuo PLA filament with Prusa Mk3) at the other end. Each composition was elaborated to maintain the density of 9% TMD (approximately 460 mg). The scheme of the set-up used is presented below (Figure 11).

Each test was prepared by mounting the produced charge between steel mounting plates. The plate located on the side of the collimator was equipped with an opening for conducting ignition with a laser pulse (520 nm, 700 mW, 100 ms). Perpendicular to the charge axis, the Phantom 9.1 high-speed camera was placed. The camera recorded the course of the combustion process with 110,000 samples per second frequency. The combustion velocity was calculated on the base of image analysis. The tests for each composition were repeated at least five times.

### 3.8. Open Air Combustion Tests

**Figure 12:**
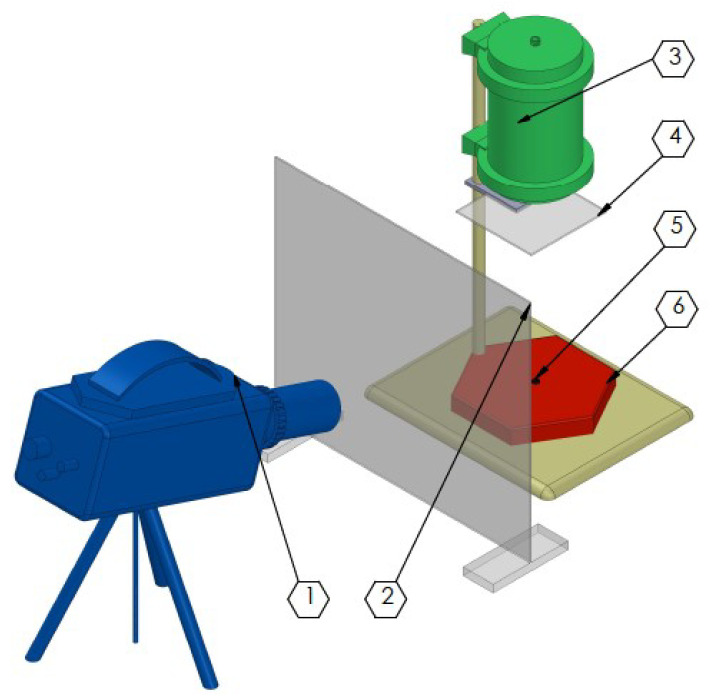
Scheme of open-air test set-up, where 1—high-speed camera; 2—PMMA shelter, 3—optical collimator, 4—PMMA shelter, 5—tested sample, 6—basic plate.

To assess the reactivity of tested compositions, a test of combustion behaviour of the materials in open air was used. The scheme of open-air test arrangement is presented below (Figure 12).

Open air combustion test was performed in the laboratory for constant temperature (20 °C) and humidity (45%). Before testing, 15 mg samples of the tested compositions were dried (60 °C, 12 h). In each test, the pre-weighted sample was placed on a ceramic baseplate, below the optical collimator protected with a 2 mm thick PMMA window. Once placed, each composition was ignited with a laser pulse (700 mW, 100 ms) generated by laser pulse generator Laser Green520-1000-SMA (Spectralaser, Poland). At the beginning of each measurement, the laser power was tested with the MIER-4W-USB thermal radiation power detector (SpectraLaser, Poland). The combustion process was recorded using Phantom 9.1 high-speed camera, operating at 6400 frames per second. In order to protect the camera lens, a 2 mm thick PMMA plate was placed in front of the camera.

## 4. Conclusions

All four tested secondary energetic materials are compatible with the Ti/CuO nanothermite system, with their choice strongly affecting both the morphology and properties of the investigated NSTEX formulations. Interestingly, while neither of the secondary energetic materials are sensitive to laser irradiation, all four NSTEXs show some degree of sensitivity. While this sensitivity is small enough not to be a safety issue, it is entirely sufficient for the development of laser-initiated pyrotechnic devices, such as igniters.

The tunability of properties of the NSTEX formulations extends also to their other properties, such as sensitivity to mechanical stimuli, which roughly corresponds to that of the utilised secondary energetic materials. The addition of nanothermite increases the sensitivity of the NSTEXs to friction while lowering their impact sensitivity in comparison with the used secondary energetic materials.

The key feature of the investigated NSTEX formulations is their ability to undergo rapid and stable combustion, without undergoing transition to detonation. In this regard, while extremely high linear combustion velocity values, ranging from 785 (NT-N) to 1092 m/s (NT-H), were recorded for the investigated NSTEX formulations, no significant correlation of combustion velocity to the velocity of detonation of the secondary energetic material was observed. The described properties of the developed NSTEXs make them highly competitive materials for practical civil and military applications.

## Figures and Tables

**Table 1:** Mechanical sensitivity of tested compositions.

Sample Name	NT-P	NT-R	NT-H	NT-N	PETN	RDX	HMX	NTO
Impact sensitivity [J]	5	50	50	50	3	3	5	40
Friction sensitivity [N]	64	192	84	84	60	120	96	360

**Table 2:** Content of tested compositions.

Sample Name	Ti wt. %	CuO wt. %	NC wt. %	EM wt. %
NT-N	25	59	1	15 (NTO)
NT-R	25	59	1	15 (RDX)
NT-H	25	59	1	15 (HMX)
NT-P	25	59	1	15 (PETN)

**Figure A5:**
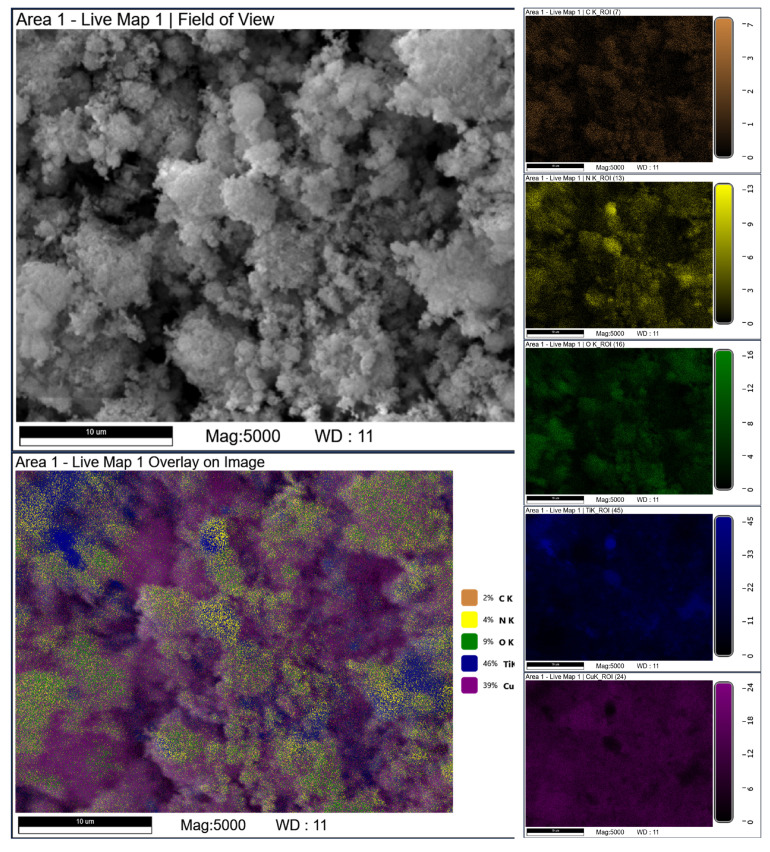
SEM-EDS images of NT-N composition.

**Figure A6:**
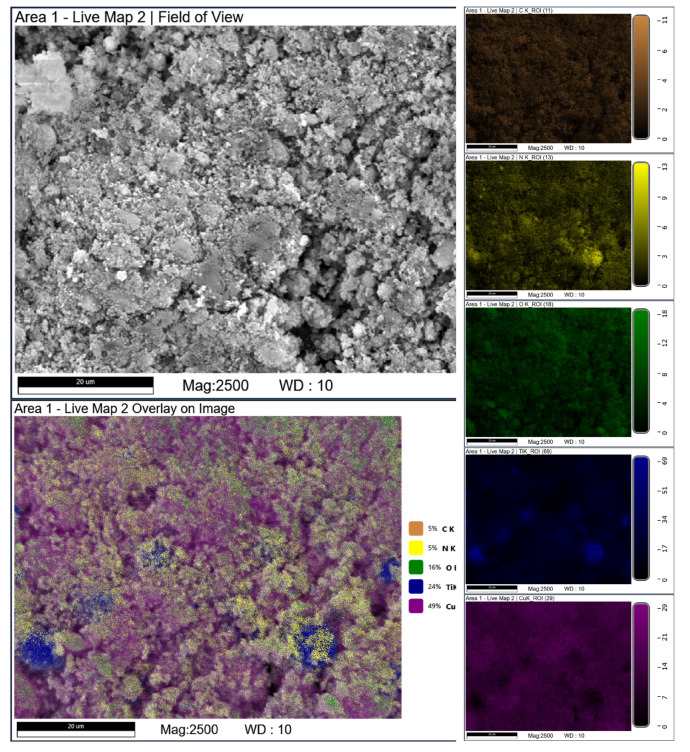
SEM-EDS images of NT-H composition.

**Figure A7:**
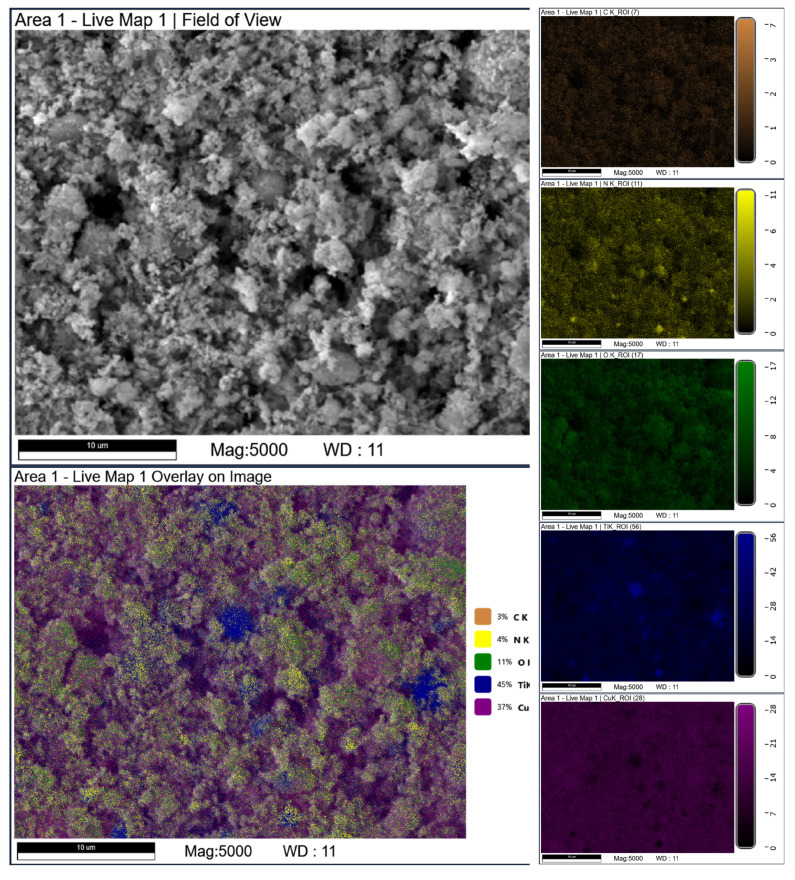
SEM-EDS images of NT-P composition.

**Figure A8:**
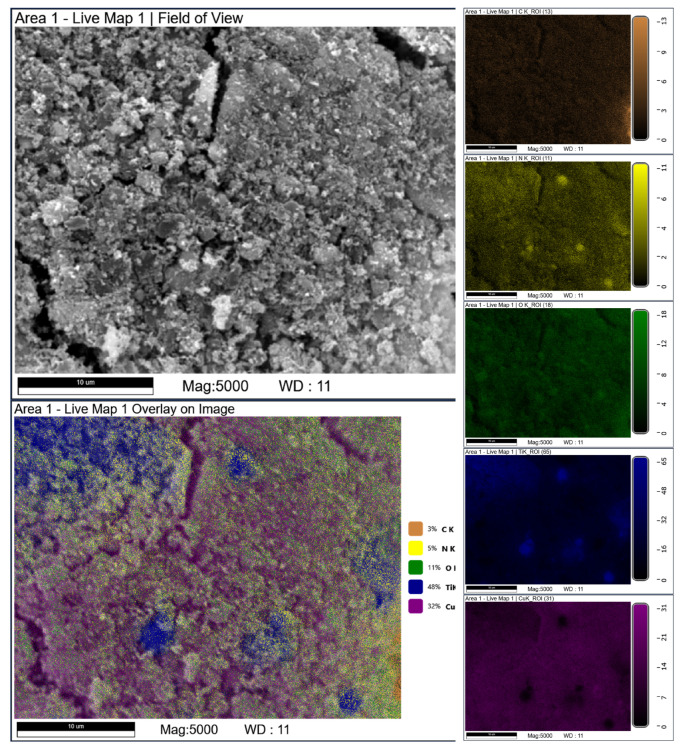
SEM-EDS images of NT-R composition.

**Figure A9:**
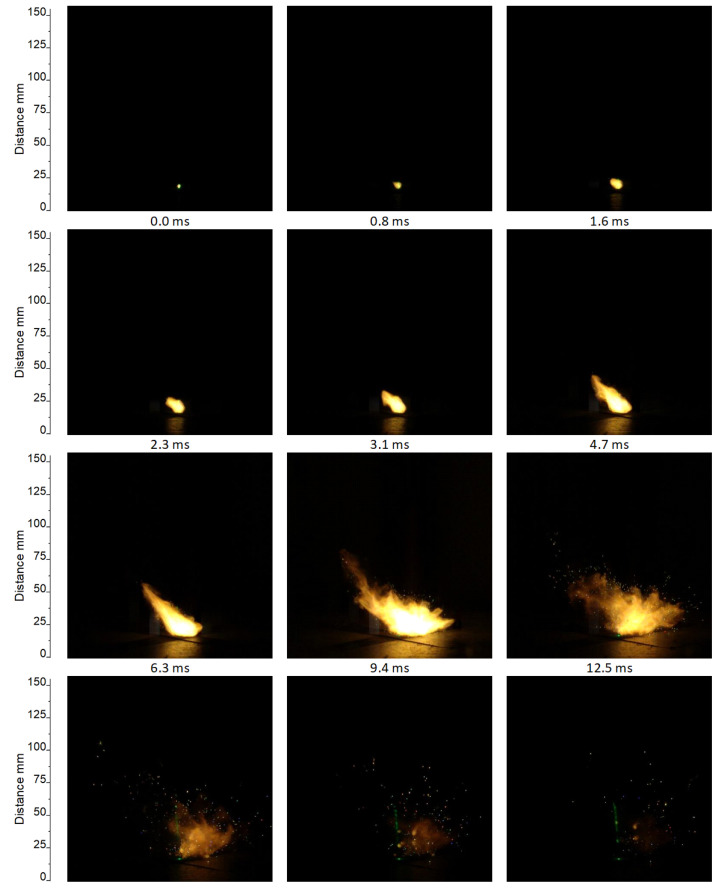
Snapshot of the open air combustion of NT-R sample.

**Figure A10:**
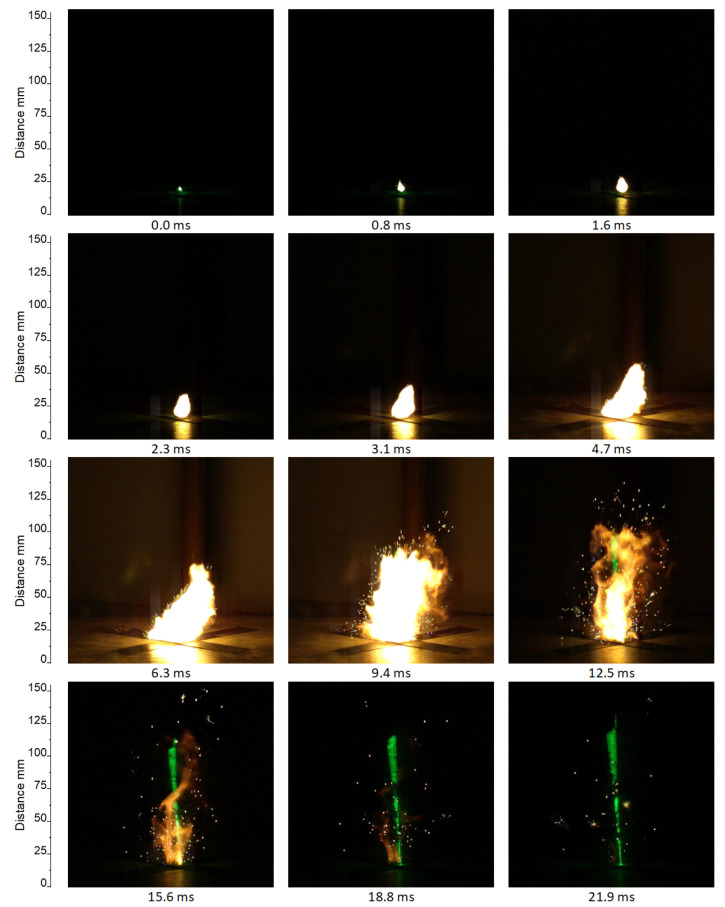
Snapshot of the open air combustion of NT-P sample.

**Figure A11:**
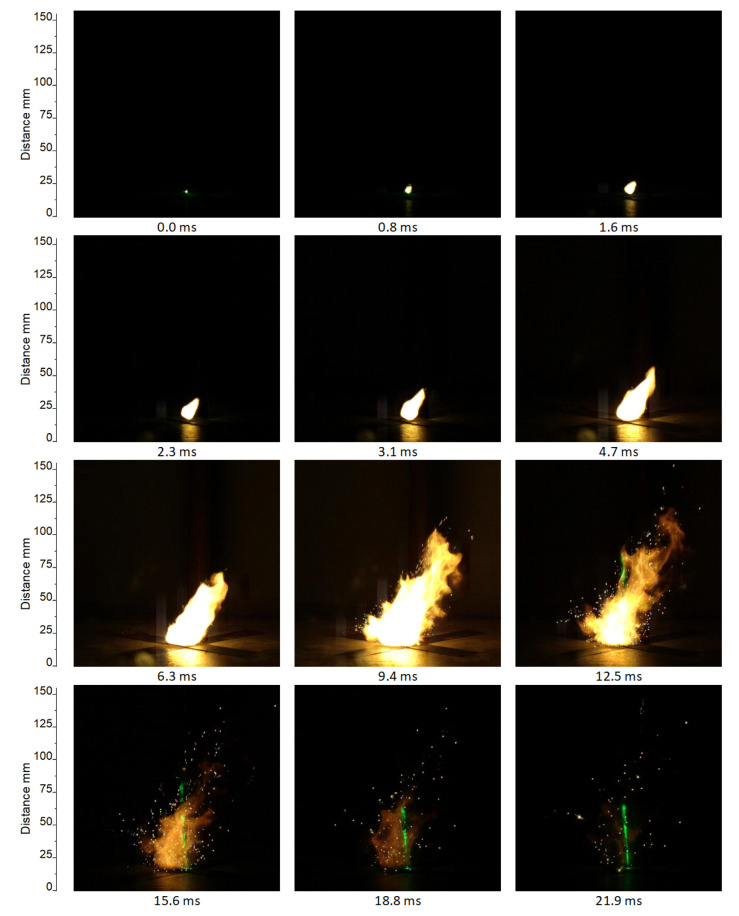
Snapshot of the open air combustion of NT-H sample.

**Figure A12:**
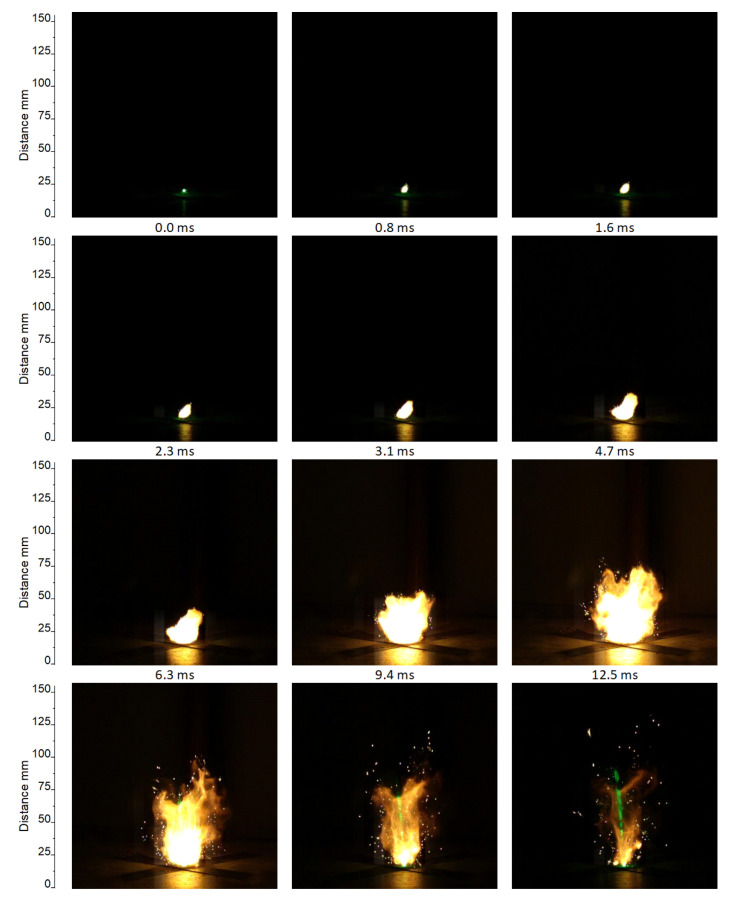
Snapshot of the open air combustion of NT-N sample.

## Data Availability

The data presented in this study are available on request from the authors.

## Abbreviations

The following abbreviations are used in this manuscript:

EM	Energetic material
HMX	1,3,5,7-Tetranitro-1,3,5,7-tetrazocane
MIC	Metastable intermolecular composites
NC	Nitrocellulose
NT	Nanothermite
NT-H	Nanothermite doped with HMX
NT-N	Nanothermite doped with NTO
NT-P	Nanothermite doped with PETN
NT-R	Nanothermite doped with RDX
NSTEX	Nanostructured thermites and explosives
NTO	5-Nitro-1,2-dihydro-3H-1,2,4-triazin-3-one
PETN	2,2-Bis[(nitrooxy)methyl]propane-1,3-diyl dinitrate
RDX	1,3,5,-Trinitro-1,3,5-triazinane
TNT	2,4,6-trinitrotoluene
